# Development of Novel Polyamide-Imide/DES Composites and Their Application for Pervaporation and Gas Separation

**DOI:** 10.3390/molecules26040990

**Published:** 2021-02-13

**Authors:** Alexandra Pulyalina, Valeriia Rostovtseva, Ilya Faykov, Maksim Tataurov, Roman Dubovenko, Sergey Shugurov

**Affiliations:** Institute of Chemistry, Saint Petersburg State University, Universitetskiy pr. 26, 198504 Saint Petersburg, Russia; v.rostovtseva@spbu.ru (V.R.); st022544@student.spbu.ru (I.F.); st022543@student.spbu.ru (M.T.); st062444@spbu.ru (R.D.); s.shugurov@spbu.ru (S.S.)

**Keywords:** polymer composites, deep eutectic solvents, polyamide–imide, pervaporation, gas separation

## Abstract

Novel polymer composites based on polyamide–imide Torlon and deep eutectic solvent (DES) were fabricated and adapted for separation processes. DES composed of zinc chloride and acetamide in a ratio of 1:3 M was first chosen as a Torlon-modifier due to the possibility of creating composites with a uniform filling of the DES through the formation of hydrogen bonds. The structure of the membranes was investigated by scanning electron microscopy and X-ray diffraction analysis; thermal stability was determined by thermogravimetric analysis and mass spectrometry. The surface of the composites was studied by determining the contact angles and calculating the surface tension. The transport properties were investigated by such membrane methods as pervaporation and gas separation. It was found that the inclusion of DES in the polymer matrix leads to a significant change in the structure and surface character of composites. It was also shown that DES plays the role of a plasticizer and increases the separation performance in the separation of liquids and gases. Torlon/DES composites with a small amount of modifier were effective in alcohol dehydration, and were permeable predominantly to water impurities in isopropanol. Torlon/DES-5 demonstrates high selectivity in the gas separation of O_2_/N_2_ mixture.

## 1. Introduction

There is a strong urge to adopt sustainable and resource-saving technologies given the current ecological context. Membrane separation methods have gained significant attention in numerous fields that consider low energy consumption, environmental friendliness, and small footprints [1].

The selection of membrane material can be identified as a critical issue in increasing the operational efficiency of the process. Polymers are evaluated as the most efficient materials for diversity, high mechanical stability, and simple preparation [2]. Hitherto, an extensive range of polymers has been employed in the membrane separation of liquid or gas mixtures, included cellulose acetate, polyvinyl alcohol, polyphenylene oxide, polyimides, etc. [3,4]. However, most research works bring out the trade-off issue between permeability and selectivity during the process [5,6]. It should be noted that one of the prospective ways to produce highly effective membrane materials is to create polymer composites [7,8]. A favorable combination of matrix and filler allows one not only to mix the properties of the composite components, but also to obtain new valuable properties.

Ionic liquids (ILs) have been recently established as a promising additive for polymer composites to improve gas separation and pervaporation processes [9]. IL is a salt consisting of an organic cation and an organic/inorganic anion, which melts at less than 100 °C. Despite a remarkable enhancement of permeability and the effect on morphology, some challenges remain in using ILs [10]. The primary limitations experienced in IL membrane preparation are the costly multi-staged synthesis and the required recycling step due to ILs’ high toxicity [11]. Furthermore, some studies declare such drawback as a deterioration of mechanical strength with the addition of ILs [12,13]. ILs have the most significant impact on the pervaporation membrane, owing to the swelling and leaching of liquid [14,15].

Deep eutectic solvents (DESs), discovered by Abbott et al. [16], draw much attention in various research fields as an alternative to ILs. DESs (similar to ILs) have some general advantages over common solvents, such as very low vapor pressure, the relatively wide temperature range of their liquid state, incombustibility, etc. In addition, in comparison with ILs, DESs are characterized by simplicity of preparation, environmental friendliness, low price, the availability of their constituent components, and the absence of the need for purification, which makes them promising as a replacement for traditional organic solvents. It has also been established in the literature that such modifiers have a plasticizing effect [17,18], which can increase productivity in the purification and separation of target components by membrane methods. Recently, DESs have proven to be a promising modifier of the polymer matrix in water treatment processes, such as ultrafiltration and nanofiltration [19,20,21]. It was shown that a DES additive provided an enhanced pore structure, resulting in both high pure water permeability and protein rejection [22]. Besides this, DESs were successfully applied in the preparation of supported liquid membranes for olefins/paraffins gas separation, which exceeded the performance of reported polymeric membranes [23,24,25].

As far as the authors are aware, there are practically no published examples of DES implementation in pervaporation processes. Thus, the task of the investigation was to estimate the influence of DESs as fillers on transport properties in pervaporation, and to continue studies in gas separation.

Pervaporation (or vapor permeation through the membrane) as an optimal membrane process for the highly selective separation of liquid mixtures is of particular interest [26,27]. The mass transport of liquid molecules through a non-porous film occurs due to selective sorption on the film surface, selective diffusion through a film, and desorption from another film side.

The pervaporation of a water–isopropanol (IPA) mixture was carried out to investigate the transport properties of Torlon and Torlon/DES composites. IPA is a widely used organic solvent in the chemical industry, and a disinfectant in pharmaceuticals [28]. In view of the peculiarities of production, the alcohol must be purified from water impurities. Pervaporation is an energy- and resource-saving method that allows one to effectively separate even small amounts of contaminants at a moderate temperature.

The membrane gas separation method allows for carrying out the continuous, reagentless, and harmless separation of gases, or obtaining a mixture enriched with one component using the available compact equipment [29]. The most critical areas of application in various industrial processes include the purification of natural gas from impurities of hydrogen sulfide, carbon dioxide, water and nitrogen; the extraction of methane from natural gas as an economically viable energy source; the oxygen enrichment of air in medicine; the obtaining of highly pure hydrogen as an alternative fuel [30].

**Figure 1:**
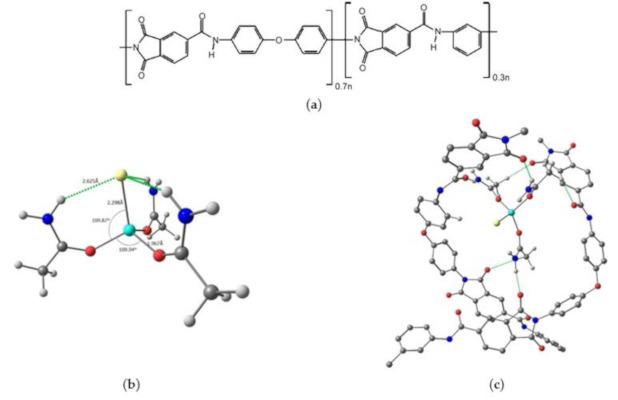
Structural formulas (**a**) PAI Torlon, (**b**) DES composed of ZnCl_2_-acetamide (1:3) and (**c**) Torlon/DES composite.

In this study, the DES composed of zinc chloride and acetamide in a 1:3 M ratio was included in polyamide–imide (Torlon), known as a promising membrane material. Polyamide–imide (PAI) is utilized extensively for membrane processes, as it exhibits high selectivity and optimal mechanical stability [31,32]. It is characterized by a relatively rigid structure and high hydrophilicity due to the presence of an amide group (Figure 1). Previously, it has been reported that PAI is applicable in alcohol dehydration, showing modest water selectivity [33,34]. Some studies have also demonstrated excellent gas separation performance [35,36]. Several approaches have been proposed for membrane stability improvement and separation efficiency.

This research aimed to examine the influence of a DES addition on the structural, physicochemical, and transport properties of a PAI (Torlon)/DES composites. A comprehensive study of the DES effect was carried out using advanced methods (X-ray, thermogravimetric analysis (TGA), mass spectrometry, scanning electron microscopy (SEM), contact angles measurement). Transport properties were investigated via the pervaporation of a water–isopropanol (IPA) mixture and gas permeation.

## 2. Results and Discussion

The study of the DES and Torlon/DES composite’s configuration was performed by quantum chemical calculations. DES (composed of zinc chloride and acetamide in a 1:3 M ratio, Figure 1b) is formed via the covalent bonding of the zinc cation and acetamide, and is soluble in amide solvents, which are used for Torlon membrane formation. The bond lengths are as follows: Zn–Cl—2.298 Å, Zn–O—1.967 Å. The angles are O–Zn–Cl—109.87°, and O–Zn–O—109.04°. Besides this, the selected DES is capable of forming hydrogen bonds with Torlon, which determines the possibility of its uniform distribution in the polymer matrix. Figure 1c demonstrates possible options for the hydrogen bond formation obtained by quantum chemical calculations. Thus, a hydrogen bond can be formed between the oxygen of the carbonyl group of the imide cycle, or of the primary amide group of the polymer, and the hydrogens of the methyl group or the amino group of acetamide.

### 2.1. Membrane Structure

**Figure 2:**
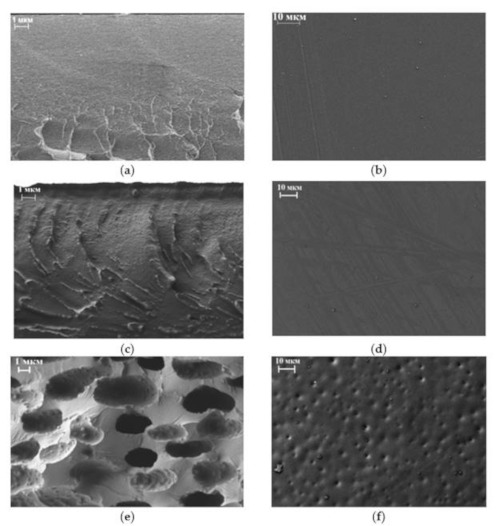
SEM micrographs of membrane surface (**b**,**d**,**f**) and cross-section (**a**,**c**,**e**): Torlon (**a**,**b**); Torlon/DES-5 (**c**,**d**); Torlon/DES-50 (**e**,**f**).

The morphology of the Torlon/DES (0–50%) membranes was examined by SEM at high magnifications. Figure 2 shows micrographs of the surfaces and cross-sections of Torlon/DES membranes containing 0, 5, and 50 wt.% DES. No apparent defects were found for the composites. Pure Torlon exhibits a fully dense and homogeneous structure (Figure 2a,b). As seen in Figure 2c,d, DES molecules are well dispersed in the polymer matrix. We observed no inhomogeneous areas or DES cluster formations in the membrane structure after DES loading up to 20%. There are no significant interfacial voids in the membrane with 5% loading. The SEM micrographs of Torlon/DES (10–20%) look similar, with no observed effects. As the DES loading increases to 50%, the DES clusters formation becomes apparent and manifests in spherical domains in the cross-sectional structure (Figure 2e). These structural changes can contribute to the enhanced permeability of the membrane.

### 2.2. XRD Analysis

**Figure 3:**
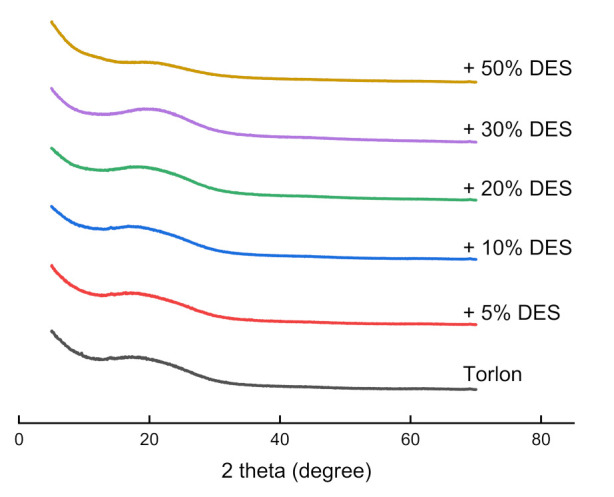
XRD patterns of pure Torlon and Torlon/DES (5–50%) composites.

The structures of the Torlon and Torlon/DES composites were characterized by XRD (Figure 3). In the case of pure Torlon, the polymer structure contains mainly an amorphous phase. The X-ray pattern shows only a broad peak at 2θ~19°. No noticeable changes can be observed for DES loading less than 20%. In this case, the d-spacing values remain almost the same, at around 4.5 Å. As the loading increases up to 50%, the intensity of peak 2θ~21° decreases with 4.8 Å d-spacing, which may indicate a decrease in the fraction of the crystalline phase in the polymer. This may be assigned to the high affinity of acetamide for the Torlon polymer matrix, and the hydrogen bond formation between them. Additionally, the plasticization effect of DES revealed a looser chain packing, and facilitated the motion of polymer segments. This behavior will be conducive to the diffusion of penetrants through the membrane.

### 2.3. Thermal Analysis

**Figure 4:**
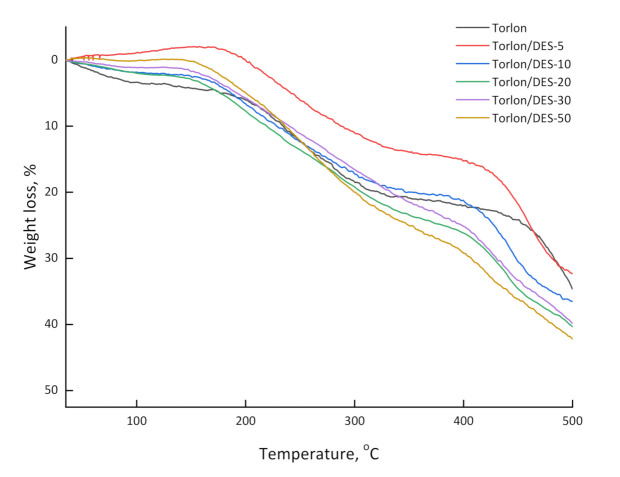
TG curves of pure Torlon and Torlon/DES (5–50%) composites.

The TGA results for the pure Torlon and Torlon/DES membranes are shown in Figure 4. Three weight loss ranges are observed for pure Torlon and the Torlon/DES composites. At the initial stage, up to 100 °C, the weight loss of the samples is about 5 wt.%, which could be attributed to the evaporation of moisture and residual solvent. In the case of composites containing acetamide as donors of the DES hydrogen bond, a further weight loss of about 14–28 wt.% is achieved with heating up to 380 °C. This is related to the decomposition of DES, as well as the removal of the residual solvent (NMP). When the samples are heated above 410–420 °C, a significant weight loss is observed, caused by the thermal destruction of the polymer and composites. 

### 2.4. Mass Spectrometry

**Figure 5:**
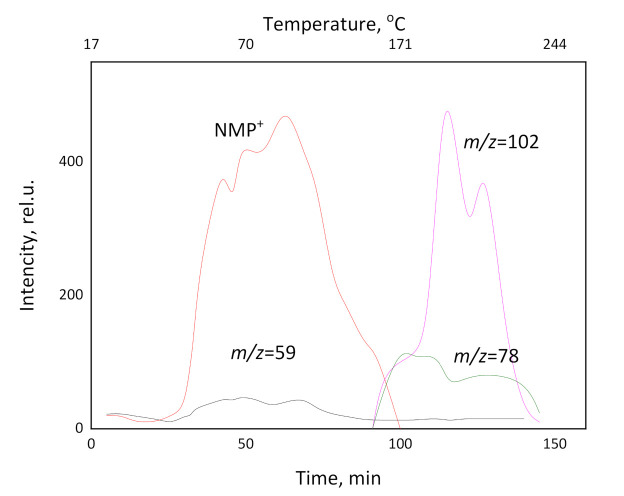
Temperature- and time-dependences of intensities of ion currents for Torlon/DES-10.

The mass spectrometric analysis of the vapor composition over the samples of Torlon/DES-10 was carried out to confirm the evaporation of NMP in the processes of heating the polymer films, to correlate weight loss with vapor components, and to find out DES components. The velocity of heating was approximately 3 K/min. The dependence of ion current vs. temperature and experiment time is presented in Figure 5. Additionally, ions from the atmospheric background (N_2_^+^, O_2_^+^, CO_2_^+^, Ar^+^) were detected in mass spectra. The peak of the mass spectrum with *m*/*z* = 99 corresponds to the direct ionization of NMP. The peak with *m*/*z* = 59 may correspond to the direct ionization of the acetamide molecule or the dissociative ionization of NMP. At the beginning of the experiment, up to 105 °C, only the atmospheric background ions were detected in the mass spectrum. In the temperature range from 105 °C to 305 °C, the ions with *m*/*z* = 59 and with *m*/*z* = 99 were detected. This correlates with the TG curve, in which the main weight loss corresponds to the interval 130–300 °C. Because the ratio between the intensities of ion currents of *m*/*z* = 59 and *m*/*z* = 99 is approximately constant during the experiment, we conclude that the origin of the ion with *m*/*z* = 59 is not the direct ionization of acetamide, but the dissociative ionization of NMP. In addition, other ions that correspond to NMP mass spectrum were detected. As NMP^+^ begins to fall to the background, new ions with *m*/*z* = 78 and 102 are produced. These ions are produced from the products of the thermal decomposition of the film. The weight loss on the TG curve above 400 °C corresponds to the thermal decomposition of the film. After the experiment, the sample of the film was extracted from the cell. It became darker and more fragile. This fact confirms the thermal destruction of the films.

From the data obtained by TGA and mass spectrometric analysis, it follows that DES loading does not affect the thermal stability of the membranes. The evaporation of residual solvent (NMP) begins from both pure Torlon and the Torlon/DES composite at about 130 °C, according to the obtained data. The evaporation of the most volatile component of DES, acetamide, occurs almost simultaneously. This follows from the fact that the weight loss in this range of the TG curve of Torlon/DES is approximately 6% higher compared to the pure film. Meanwhile, the mass spectra of the vapor composition over the modified membrane exhibit peaks corresponding to the ionization of acetamide. Polymer thermal destruction for both membranes begins at 360 °C. The second component of DES, ZnCl_2_, evaporates at higher temperatures (~700 °C) [37,38], which are beyond the investigated temperature range.

### 2.5. Contact Angles

Contact angle measurements were conducted to estimate the effect of a DES filler on the hydrophilicity of the polymer matrix. Table 1 shows data on the contact angles of the water and ethylene glycol of the Torlon/DES membranes containing 0–50 wt.% DES. Torlon is considered to be a hydrophilic polymer since the contact angle of water is less than 90°, which is in good agreement with previously reported data [33]. With the increase in DES content, the contact angle of water and ethylene glycol gradually decreases. 

**Figure 6:**
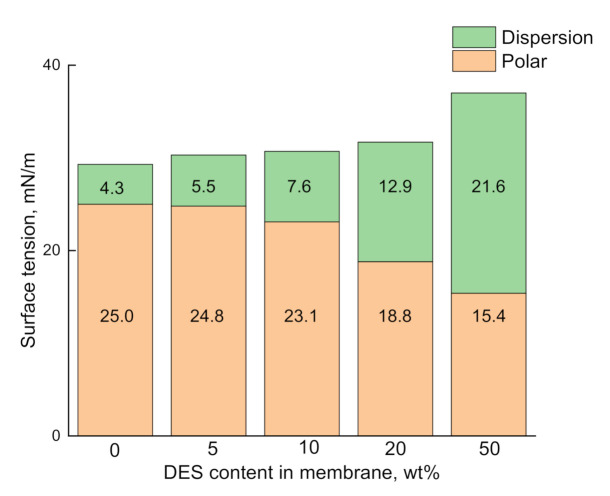
Dependence of the surface tension on DES content in the Torlon/DES membranes.

The values of contact angles were used to calculate the polar and dispersion contributions of surface tension, employing the Owens–Wendt method [39]. The results are given in Figure 6. As the loading of DES increases, the dispersion contribution increases, while the polar one decreases. As such, loading DES onto the polymer matrix brings about an increase in the surface hydrophilicity of the polymer composites.

### 2.6. Pervaporation

**Figure 7:**
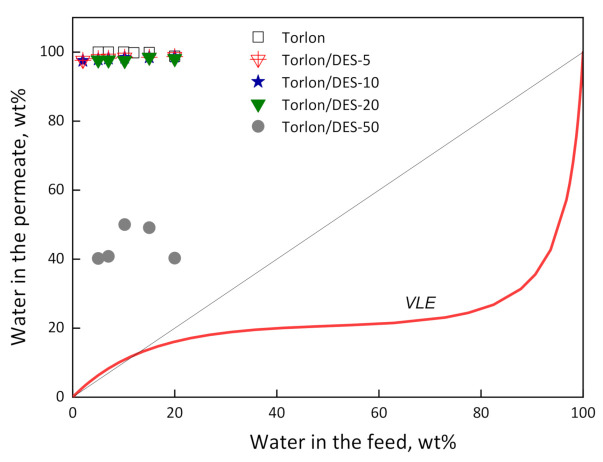
Dependence of water concentration in the permeate on water concentration in the feed for pervaporation using Torlon-based membranes; the vapor–liquid equilibrium (VLE) curve for water–IPA mixture, 50 °C.

**Figure 8:**
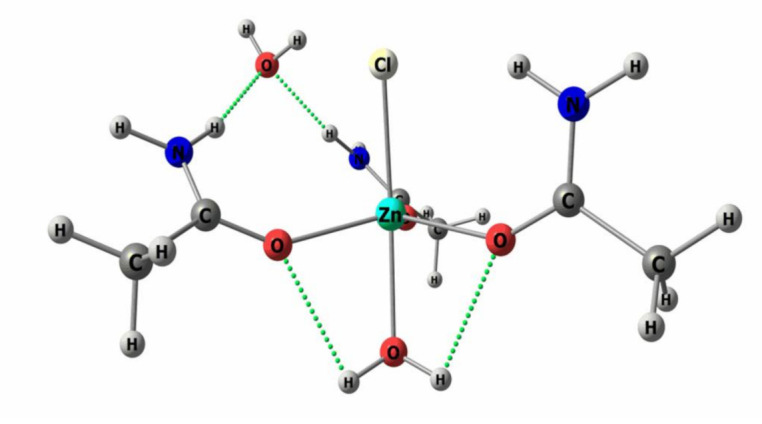
Scheme of DES and water coordination.

The separation of a water–IPA mixture by pervaporation was studied over a wide concentration range of feed mixtures for composite membranes Torlon/DES at 50 °C. Figure 7 illustrates the dependence of the water content in the permeate on the water content in the feed, and the vapor–liquid equilibrium curve for the water–IPA mixture. It appears that all membranes show high selectivity towards water. In the range of 5–20 wt.% water in the feed, the permeate was enriched with water (more than 97 wt.%), except for the Torlon/DES-50. The selective transport of water molecules even with DES inclusion can be explained by an additional interaction of zinc ions and acetamide with water via complexation and hydrogen bonds (Figure 8).

The lower concentration of water in the permeate for Torlon/DES-50 in comparison with pure Torlon is attributed mainly to significant changes in morphology, which is confirmed by the SEM results. DES clusters were formed in the structure, including regions with increased permeability for both water and alcohol. 

**Figure 9:**
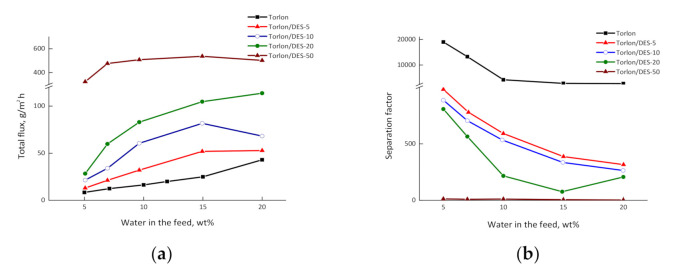
Dependences of (**a**) the total flux and (**b**) the separation factor on water concentration in the feed for the pervaporation of water–IPA mixture using the Torlon-based membranes at 50 °C.

Figure 9 shows the dependencies of the total flux and the separation factor on the water concentration in the feed for the pervaporation of the water–IPA mixture using the Torlon-based composites. The introduction of up to 20 wt.% DES leads to a noticeable increase in the total flux of the composites, as compared to pure Torlon. With an increase in the water content in the feed, an increase in the total flux for all composites occurs. The maximal total flux belongs to the Torlon/DES-50 composite, and is more than ten times higher than that of Torlon. This enhancement is ascribed mainly to the looser structure and higher hydrophilicity of the composite membrane. 

On the other hand, the separation factor decreases with the increasing DES content in the membrane and water concentration in the feed. This behavior has a similar cause: the increased hydrophilicity and special voids caused by DES, which are beneficial to the sorption and diffusion of both water and IPA molecules in the membranes.

The Torlon/DES-50 membrane has a higher permeability than the others, but the separation selectivity is dramatically lower. 

#### Comparison with Literature Data

A comparison of the Torlon/DES composite’s performance with the reported polyheteroarylene membranes is presented in Table 2 for IPA dehydration. It can be highlighted that the Torlon/DES composite showed a comparable flux and good selectivity for removing water from IPA at relatively low temperatures. 

Among all, polyimides with various additives possess superior pervaporation properties [45,46,47,48]. The inclusion of DES in the Torlon matrix results in an increased performance compared with the Torlon film. Therefore, DES could be regarded as an additive to a polymer matrix, as it can facilitate mass transport through the membrane.

### 2.7. Gas Transport Properties

**Figure 10:**
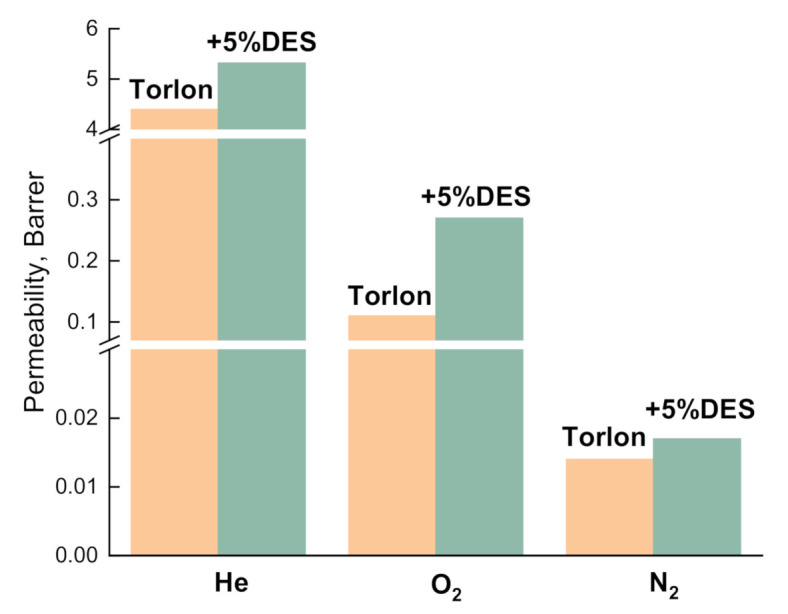
Permeability of Torlon and Torlon/DES-5 composites for He, N_2_, and O_2_ at 30 °C.

The gas separation properties of the Torlon and Torlon/DES-5 membranes were studied via the barometric method for individual gases: He, N_2_, O_2_. The Torlon/DES-5 composite was chosen as the object of the study because the inclusion of 5 wt.% of DES leads to the highest selective properties compared to other Torlon/DES composites. Table 3 summarizes the performances of membranes in terms of permeability and ideal selectivity. The DES loading leads to an increase in permeability coefficients for all gases (Figure 10). It is mainly attributed to the enlarged free volume of the modified membrane due to the rearrangements of polymer chains and the more amorphous structure. The selectivities of the He/O_2_ and He/N_2_ gas pairs show a decreasing tendency with an increase in DES loading. On the other hand, O_2_/N_2_’s selectivity slightly increases after DES inclusion.

#### Comparison of Gas Separation Performance

**Figure 11:**
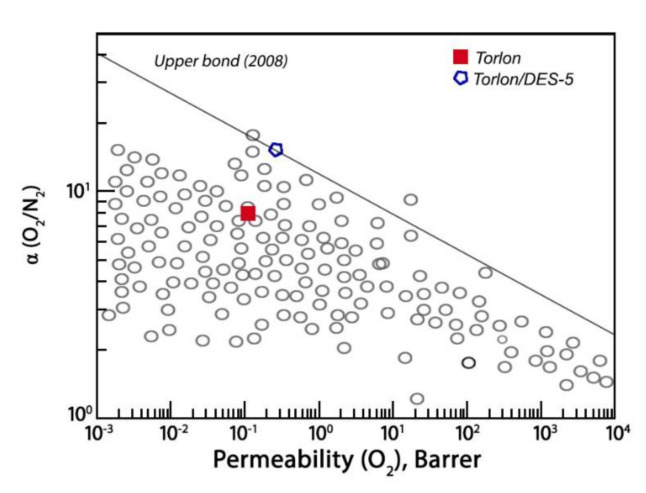
Oxygen/nitrogen selectivity as a function of oxygen permeability. The upper bound line was taken from Robeson’s diagrams [49].

The performances of Torlon-based membranes were compared with reported polymer membranes. The data on the O_2_ permeability and O_2_/N_2_ selectivity of Torlon and the Torlon/DES-5 composite were plotted on the well-known Robeson diagram [49]. As seen from Figure 11, the membranes under study are effective for O_2_/N_2_ separation. The position of the Torlon/DES-5 composite is around the Robeson upper bound for O_2_/N_2_ separation. This fact indicates that the addition of DES to the polymer matrix has the potential to improve the gas transport properties of the membrane when applied in the separating of individual gases from the air and obtaining gas mixtures for medical purposes.

## 3. Materials and Methods

### 3.1. Materials

PAI Torlon^®^ 4000TF, M_n_ = 30,000 g/mole, Т_g_ = 285 °C was purchased from Solvay (Solvay Specialty Polymers, Brussel, Belgium). *N*-methyl pyrrolidone (NMP), ethylene glycol, isopropanol, zinc chloride (ZnCl_2_), and acetamide (AcA) of chemically pure (CP) grade were purchased from Vekton (Vekton, Saint Petersburg, Russia) and were used as received. All gases for permeation tests were supplied by Atmosphere (Atmosphere, Saint Petersburg, Russia) at a minimum purity of 99 wt.%.

### 3.2. Membrane Preparation

Membranes were prepared by the solution casting and solvent evaporation method from Torlon solution in NMP containing various amounts of DES (5–50 wt.%). At compositions above 50% DES it was not possible to obtain a homogeneous polymer film with mechanical properties that would be adequate for testing in pervaporation. A 12% polymer solution was prepared by dissolving Torlon in NMP under stirring for 2–3 h at 60 °C. Then, the solution was filtered and degassed using a sonic bath (Sapphire, Moscow, Russia). 

Zinc chloride and acetamide in 1:3 M ratios were mixed at 70 °C on a heat plate with magnetic stirring. The DES was considered formed when the mixture became transparent. 

A certain amount of DES was added to the polymer solution with stirring and sonication for 40 min. After that, the Torlon/DES solution was cast on a Petri dish. The obtained membranes were dried in a conventional oven at 70 °C for two days. After that, the membranes were subjected to vacuum drying at 60 °C to a constant weight. The thicknesses of the Torlon/DES membranes were 40–50 μm.

### 3.3. Computational Methods

Thermodynamically stable conformations of molecules were pre-estimated using ChemOffice CS Chem3D Ultra by methods of molecular mechanics (MM2, MMFF94) and molecular dynamics. All quantum chemical calculations presented in this study were performed using Gaussian 09W software and visualized using Chemcraft. The molecular structures were optimized and characterized using the unrestricted Hartree–Fock (UHF) theory with 6-31G and 6-31G* basis sets.

### 3.4. Membrane Characterization

The surfaces and cross-sections of the membranes were studied with a Zeiss SUPRA 55VP scanning electron microscope (SEM) (Carl Zeiss, Oberkochen, Germany) equipped with In-lens SE and SE2 secondary electron detectors, a secondary electron detector for low vacuum mode (VPSE), and a four-quadrant backscattered electron detector (AsB). Samples were coated with a 20 nm thick carbon layer using the Quorum 150 cathode sputtering installation (Quorum Technologies Ltd., Lewes, UK) prior to the experiment. For investigating the membrane cross-sectional morphology, the membranes were first cracked in liquid nitrogen, coated with carbon, and then observed using scanning a electron microscope via the same equipment.

X-ray diffraction (XRD) analysis was performed on an X-ray diffractometer D8 DISCOVER (Bruker, Bremen, Germany) equipped with a CuKa radiation source with a wavelength of 1.54 Å. The scans were made with a step size of 0.058, ranging from 5° to 50°.

The thermal stability of Torlon/DES membranes was examined via a TG 209 F3 Iris thermo-microbalance (Netzsch, Selb, Germany) at a heating rate of 10 °C/min from room temperature to 500 °C in a nitrogen atmosphere. 

The mass spectrometric investigation was carried out using the Knudsen effusion technique combined with mass spectrometric analysis of the vapor composition on an MS 1301 mass spectrometer (Construction Bureau, Academy of Science, Saint Petersburg, Russia). Ionization of the vapor species was done by electron ionization, with the energy of the ionizing electrons being 25 eV. The samples were evaporated from an open gold effusion cell placed in a molybdenum block and heated by a resistance furnace. The temperature was measured with a Pt−PtRh thermocouple and stabilized with an accuracy of ±1 °C.

The contact angle measurements were carried out using the sessile drop method on a DSA 10 drop shape analyzer (KRÜSS GmbH, Hamburg, Germany) at room temperature and atmospheric pressure. The liquids under study were water and ethylene glycol, with surface tension (*σ_l_*) equal to 72.4 mN/m and 47.7 mN/m, correspondingly. Based on the measured contact angles, the critical surface tension (*σ_s_*) and its components were calculated by the Owens–Wendt method [39].

(1) $$\sigma_{S}=\sigma_{S}^{d}+\sigma_{S}^{p}$$

where $\sigma_{s}^{d}$ is the dispersion component and $\sigma_{s}^{p}$ is the polar component of membrane surface tension.

### 3.5. Pervaporation

**Figure 12:**
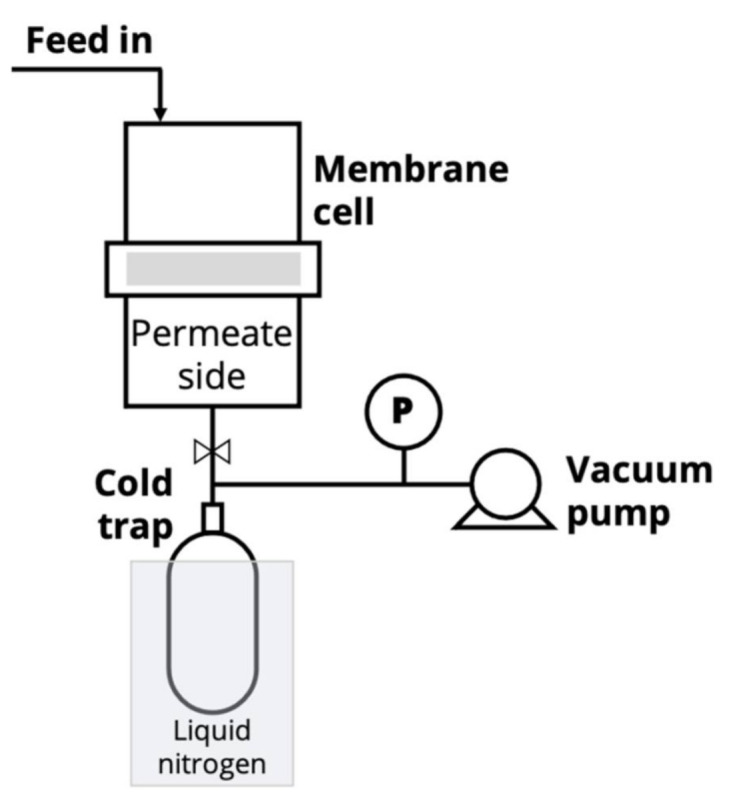
Schematic of the laboratory-scale pervaporation setup.

The pervaporation experiments were conducted via a lab-scale apparatus with stirring at 50 °C. The effective membrane area was 14.8 cm^2^ (Figure 12). Downstream pressure below 10^−2^ mm Hg was maintained on the permeate side with a vacuum pump MD 1C (Vacuubrand GMBH, Wertheim, Germany). Water–IPA mixture was used as a feed. The permeate was collected into a trap immersed in liquid nitrogen and weighted with the balance Mettler Toledo ME204 (Mettler Toledo, Columbus, OH, USA). The feed and product concentration were analyzed by a gas chromatograph «Chromatec–Crystal 5000.2» (Chromatec, Yoshkar-Ola, Russia) with a thermal conductivity detector. The experiments were repeated three times, and the average value of the results was considered.

Total flux through membrane (*J*) was determined as the amount of liquid penetrating through the membrane area per time unit. To compare membranes with different thickness (*l*) values from 40 to 50 µm, the value of total flux normalized (*J_n_*) was used. *J_n_* is the flux through the membrane, with that for a 20 µm thick membrane calculated as:

(2) $$J_{n}=\frac{J\cdot l}{20}$$

The separation factor *β_Water/IPA_* was defined according to the equation [50]:

(3) $$\beta_{Water/IPA}=\frac{Y_{Water}/Y_{IPA}}{X_{Water}/X_{IPA}}$$

where *Y* and *X* are the weight fraction of components in the permeate and feed, respectively.

### 3.6. Gas Separation

**Figure 13:**
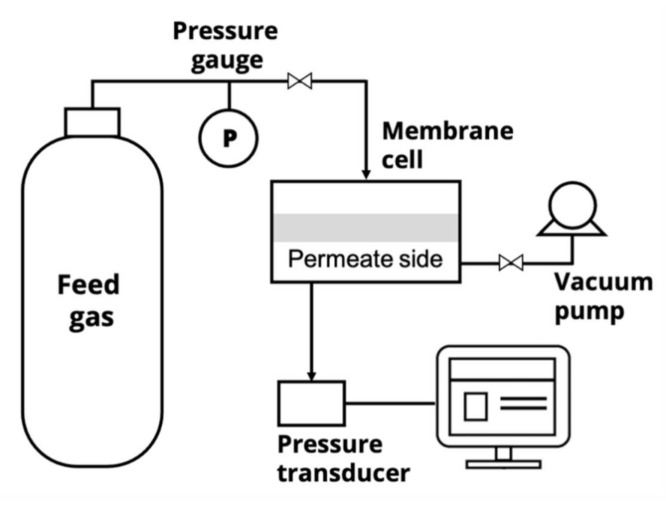
Schematic of the laboratory-scale gas permeation setup.

Gas permeation tests were performed using single gases with high purity (He, O_2_, N_2_) by the barometric technique using a laboratory high-vacuum apparatus with a static permeation cell and with an effective membrane area 5.25 cm^2^ at 30 °C (Figure 13). The membrane sample was placed and sealed in a module that was evacuated. At least two films were examined for the reproducibility test. At the beginning of the permeation experiment, the gas under constant pressure, *p* (150 kPa), was brought into the feed part of the permeation cell. The permeability was determined from the increase in pressure Δ*p* in a calibrated volume *V_p_* of the product part of the cell per the time Δ*t* interval during steady-state permeation. The gas permeability coefficient *P* was estimated by the following equation:

(4) $$P=\frac{\Delta p_{p}}{\Delta t}\cdot \frac{V_{p}\cdot l}{S\cdot p}\cdot \frac{1}{RT}$$

where *l* is the membrane’s thickness, *S* is its area, *T* is the absolute temperature, and *R* is the gas constant. All measurements were carried out at 30 °C. The permeability coefficient *P* was expressed in Barrers (1 Barrer = 10^−10^ cm^3^ (STP) cm/(cm^2^ s cmHg)). 

The ideal selectivity *α_i_*_/*j*_ for gas *i* relative to gas *j* was calculated according to the following equation:

(5) $$\alpha_{i/j}=\frac{P_{i}}{P_{j}}$$

## 4. Conclusions

Novel composites based on polyamide–imide (Torlon) modified with a modern type of filler, that is, deep eutectic solvent (DES) (composed of zinc chloride and acetamide in a 1:3 M ratio), were prepared and studied in this work. The morphology of the Torlon/DES composites was studied by SEM. The loading up to 20 wt.% DES hardly contributed to the morphology. It was shown that there are structural changes in the Torlon/DES-50 composite, which manifest as spherical domains that appear due to the formation of DES clusters. X-ray diffraction revealed the plasticization effect of DES and a decrease in the crystalline phase of the polymer matrix.

Incorporating the DES in the Torlon reduces the contact angles of water and ethylene glycol, and increases critical surface tension; this means that the Torlon/DES composite’s surface becomes more hydrophilic than pure Torlon. The data on the TGA showed that the thermal stability of the Torlon-based composites remains at an acceptable level after DES loading into the matrix.

The transport properties in the pervaporation of the water–IPA mixture were studied for Torlon/DES composites containing 0–50 wt.% DES. All the membranes were predominantly selectively permeable towards water. With increasing DES contents in the polymer matrix, the total flux through the membrane increases, but the separation factor decreases. The Torlon/DES-50 composite has a higher flux than the others, but the selectivity in the mixture separation is dramatically lower. It was found that the optimal loading is 10 wt.% DES, which allows one to achieve a comparable dehydration performance.

The gas transport properties of the Torlon/DES-5 composite were compared with those of Torlon by measuring H_2_, N_2_, and O_2_ permeation. With DES loading in Torlon, the permeability coefficients for all gases increase, whereas the selectivity in H_2_/N_2_ and He/O_2_ separation decreases, but O_2_/N_2_ selectivity slightly increases. Thus, the addition of DES to the polymer matrix has the potential to improve the transport properties of membranes.

## Figures and Tables

**Table 1:** Contact angles of Torlon/DES composites.

Membrane	Contact Angle, Degree	
	Water	Ethylene Glycol
Torlon	87.0 ± 2.1	58.4 ± 1.4
Torlon/DES-5	84.1 ± 1.9	55.6 ± 0.9
Torlon/DES-10	80.7 ± 1.7	53.4 ± 1.4
Torlon/DES-15	78.5 ± 1.6	52.4 ± 1.5
Torlon/DES-20	74.7 ± 1.7	51.1 ± 0.8
Torlon/DES-50	65.3 ± 2.1	44.5 ± 0.9

**Table 2:** Comparison of the transport properties of polyheteroarylene membranes in the pervaporation of the water–IPA mixture.

Membrane	T, °C	Water in the Feed, wt.%	Water in the Permeate, wt.%	Total Flux, g/m^2^h	Separation Factor	Ref.
6FDA-HAB/DABA polyimide	60	15	99.9	≈2000	22,500	[40]
Matrimid polyimide	100	18	99	198	575	[41]
P84 copolyimide	60	15	99.8	64	2908	[42]
Torlon 4000TF polyamide-imide	60	15	99.8	6.8	2973	[33]
Ultem 1010 polyetherimide	60	15	99.0	7	585	[33]
BPADA-ODA-DABA polyimide	60	15	≈99.5	≈40	≈1128	[43]
TC-PIO-300	60	10	99.7	≈24	≈3100	[44]
Matrimid/Zeolite 4A *	30	10	≈99.98	21	29,990	[45]
PI-0.5%NHGO-400 *	60	15	99.8	161.5	>5000	[46]
6FDA-Durene-DABA-20% FeAC-400	60	15	99.8	120	4298	[47]
Matrimid, 5% Cyclodextrin **	22.4	14	99.8	50	>5000	[48]
Torlon/DES-10	50	15	98.5	82	216	This work

*—mixed matrix membrane; **—composite membrane.

**Table 3:** Gas transport properties of membranes at 30 °C.

Membrane	Permeability, Barrer			Ideal Selectivity		
	He	O_2_	N_2_	O_2_/N_2_	He/O_2_	He/N_2_
Torlon	4.4	0.11	0.014	7.9	40	0.56
Torlon/DES-5	5.32	0.27	0.017	15.9	19.7	0.33

## Data Availability

Not applicable.
